# Trends in Mortality Rates From Hemophagocytic Lymphohistiocytosis in the United States From 2010 to 2023

**DOI:** 10.7759/cureus.103346

**Published:** 2026-02-10

**Authors:** James Di Palma-Grisi, Evan Locke, Steffen Kulp, Suraj Pothineni, Harsh Parmar

**Affiliations:** 1 Internal Medicine, Hackensack University Medical Center, Hackensack, USA; 2 Internal Medicine, Hackensack Meridian School of Medicine, Hackensack, USA; 3 Oncology, John Theurer Cancer Center, Hackensack, USA

**Keywords:** cdc-wonder, epidemiology, hematology, hemophagocytic lymphohistiocytosis (hlh), registry study

## Abstract

Hemophagocytic lymphohistiocytosis (HLH) is a severe, life-threatening condition marked by uncontrolled T-lymphocyte and macrophage-mediated inflammation, usually secondary to an underlying malignancy or infection in adults. It leads to multiorgan failure and death if not promptly recognized and treated; adult secondary HLH carries an overall mortality rate of 57% in an ICU population regardless of treatment. We examined trends in age-adjusted mortality rates from the Centers for Disease Control and Prevention's Wide-ranging Online Data for Epidemiologic Research (CDC WONDER) database to further characterize this interesting multinational finding in an otherwise rare disease. Age-adjusted mortality rates (AAMRs) from HLH showed consistent yearly increases, most of which were statistically significant, with an overall 12-fold rise from 2010 to 2023. This trend was mirrored in all census regions and in most demographic categories.

## Introduction

Hemophagocytic lymphohistiocytosis (HLH) is a hyperinflammatory syndrome that can have an insidious, nonspecific presentation but can rapidly cause multiorgan failure and death via T-lymphocyte and macrophage activation in both primary and secondary HLH if not promptly recognized and treated [1-4]. Recent registry studies from multiple countries demonstrate both rising incidence and mortality associated with HLH among adults, particularly in hospitalized populations. This trend appears specific to HLH (International Statistical Classification of Diseases and Related Health Problems 10th Revision (ICD-10 code: D76.1), as related histiocytic disorders such as Langerhans cell histiocytosis (D76.0), infection-associated HLH (D76.2), and other histiocytosis syndromes (D76.3) have not demonstrated similar increases in mortality rates [5].

In 2018, the only single year in which D76.1 and D76.2 deaths were reported, there were 281 deaths attributed to D76.1 and 10 attributed to D76.2. In light of this, in this study, we limited our analysis to the D76.1 records [2]. Whether the increase in recorded deaths reflects improved diagnostic recognition and coding, an aging population, improved survival times after treatment, or an underlying worsening disease and mortality burden associated with HLH remains unclear, with one recent study from Germany finding that most of the increase in death rates was attributable to increased rates of diagnosis among older patients [6].

Identifying the underlying condition that triggers HLH in secondary cases, which is responsible for nearly all cases in adults, is critical for effective treatment [7]. One recent study from France found that 83% of death records listing HLH lacked an underlying diagnosis, while most of the remaining cases were attributed to a known malignancy, most commonly lymphoma [8,9]. Another recent study from Sweden closely examined hospital records of 67 cases with both HLH and a malignancy listed on their death records and found that 13% were attributable to Langerhans cell histiocytosis, nonmalignant hematologic conditions, or the setting of recent allogeneic hematopoietic stem cell transplantation, with a single case of primary HLH in an adult [10]. One longitudinal study from Japan demonstrated that the incidence of HLH in adults with known underlying malignancies was low (<1%) but carried an overall mortality rate of 50% despite treatment [11]. This is comparable to a multicenter study from multiple ICU sites in France describing a 57% overall mortality rate regardless of treatment [1].

HLH can be difficult to study because of this diagnostic uncertainty, both regarding its numerous underlying conditions and because of relatively high rates of misdiagnosis related to its vague, constitutional presentation [12-14]. One recent study from England found that more than half of death records recording the D76.1, D76.2, and D76.3 groups were coded with D76.1, and in this group, 99% of records contained HLH or macrophage activation syndrome (MAS) in the free text validation summary. Nearly all D76.2 coded records (99%) contained HLH or MAS in the validation summary, while less than half of D76.3 coded records (43%) contained either diagnosis in the validation summary [15].

One recent review of death records from 29 European countries found that age-standardized death rates varied widely between countries, which the authors attributed to potential underrecognition of the condition across different practice environments [16]. To better understand these trends in the United States, we conducted a population-level analysis of age-adjusted mortality rates (AAMRs) for HLH (D76.1) from 2010 to 2023. The process by which the Centers for Disease Control and Prevention's Wide-ranging Online Data for Epidemiologic Research (CDC WONDER) database calculates the AAMR is described in the Methods section. We compared the AAMR associated with the D76.1 code to the AAMR from the remaining D76 cluster (D76.0, D76.2, D76.3).

An earlier version of this work was presented as a poster abstract at the 2025 American Society of Hematology annual meeting on December 7, 2025.

## Materials and methods

This study involves a large, retrospective cohort analysis using the publicly available death record data from CDC WONDER [2]. We identified the relevant diagnostic codes in the ICD-10 [17]. The ICD diagnostic code D76.1 for HLH was our primary code of interest, and we exported age-adjusted mortality data from 2010 to 2023 across census region, ethnicity, and sex.

Age adjustment was performed using the following formula:

AAMR = Σ i * ( Psi / Ps ) * Ri

Where AAMR is the age-adjusted mortality rate, i is the age group (in this case, in 10-year bands), Psi is the standard population for the age group, and Ps is the total U.S. standard population [18]. All AAMR calculations are presented unchanged and as provided by the CDC WONDER reporting tools.

We also exported mortality data associated with the ICD codes D76.0 (other specified diseases with participation of lymphoreticular and reticulohistiocytic tissue), D76.2 (hemophagocytic syndrome, infection-associated) and D76.3 (other histiocytosis syndromes), as well as mortality data associated with the ICD code D89.8 (other specified disorders involving the immune mechanism), which includes graft-versus-host disease (GvHD), a condition in which leukocytes present in donor tissue recognize and attack their host. We employed the "underlying cause of death" functionality available on CDC WONDER, which reports the number of death certificates with the selected ICD code as the principal cause of death [2]. We also used the standard error calculated within the CDC WONDER platform to ensure statistical significance of any observed trends across geographic region, ethnicity, and sex from 2010 to 2023. We chose to include only data from patients who were 25 years or older at the time of death to limit our study population to adults who developed secondary HLH.

## Results

**Figure 1:**
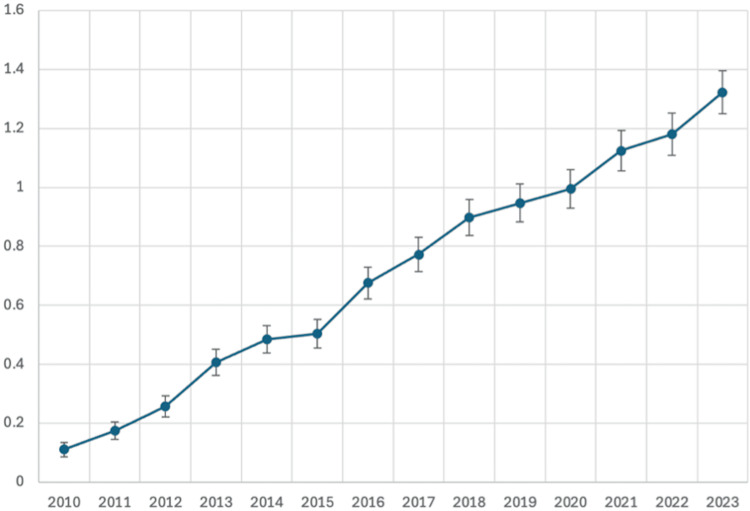
AAMR per 1,000,000 from HLH The graph shows consistent yearly increases, including throughout the years of the COVID-19 pandemic. Most year-on-year increases were statistically significant, with an overall significant twelvefold increase from 2010 to 2023. The year is presented on the X-axis, and the AAMR per 1,000,000 is presented on the Y-axis AAMR: age-adjusted mortality rate; HLH: hemophagocytic lymphohistiocytosis; COVID-19: coronavirus disease 2019

There was a significant increase in the AAMR per 1,000,000 associated with HLH from 2010 (0.1106) to 2023 (1.3224), with a steady annual rise. When stratified by census region, these trends were observed in all regions from 2013 to 2023. The AAMR increased in the Northeast from 2014 (0.5448) to 2023 (1.5969), in the Midwest from 2013 (0.5776) to 2023 (1.4584), in the South from 2013 (0.3003) to 2023 (1.1951), and in the West from 2013 (0.4295) to 2023 (1.3688) (Figure 1).

**Figure 2:**
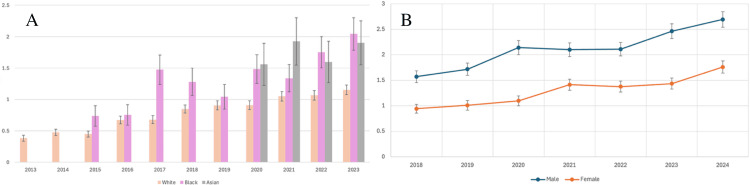
Trends classified by race and sex A (left): AAMR per 1,000,000 from HLH by ethnicity. B (right): AAMR per 1,000,000 from HLH by sex. In both charts, the year is presented on the X-axis, and the AAMR per 1,000,000 is presented on the Y-axis AAMR: age-adjusted mortality rate; HLH: hemophagocytic lymphohistiocytosis

Full data were not available for every demographic group, but we report the available consecutive data here. Males showed a significant increase from 2018 (1.5718) to 2023 (2.4634), and females showed a significant increase from 2018 (0.9433) to 2023 (1.4361). Caucasians showed a significant increase in AAMR from 2013 (0.3813) to 2023 (1.1534), African-Americans showed a significant increase from 2015 (0.7381) to 2023 (2.0433), and Asians showed a nonsignificant increase from 2020 (1.5591) to 2023 (1.9040) (Figure 2).

**Figure 3:**
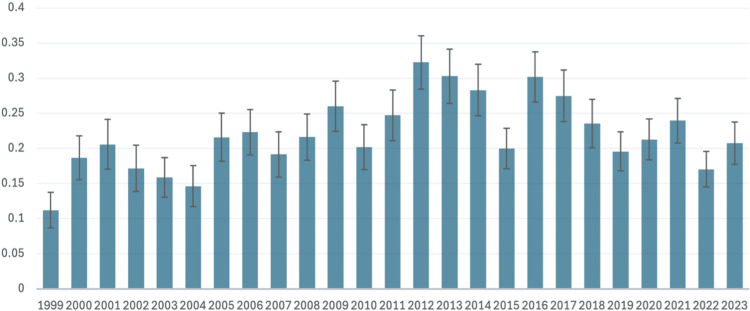
AAMR per 1,000,000 from the rest of the D76 cluster (D76.0, D76.2, D76.3) from 1999 to 2023 The year is presented on the X-axis, and the AAMR per 1,000,000 is presented on the Y-axis AAMR: age-adjusted mortality rate

The rest of the D76 cluster (D76.0, D76.2, D76.3) did not demonstrate any consistent trend (Figure 3), with a significant overall increase from 1999 (0.1121) to 2010 (0.2020) and a minimal but significant increase from 2010 to 2023 (0.2077). GvHD, itself a rarely recorded cause of death, did not show any consistent pattern from 2020 (0.195) to 2024 (0.195), the only period for which consecutive yearly data were available. Data from 2024 were provisional at the time of analysis.

## Discussion

There has been a clear rise in the AAMR from HLH across census regions, ethnicities, and sexes. Overall survival from secondary HLH remains low. Among cases secondary to an underlying malignancy, the most common primary condition, overall survival has recently been estimated at 20-30% over two years [5]. Our population-level mortality data for HLH (D76.1) were collected from 2010 to 2023, and virtually all cases would have been diagnosed after the 2004 revision of the HLH-94 criteria. We acknowledge that uptake of the revised criteria may vary by region or practice setting. The original HLH-94 criteria for primary HLH in a pediatric population required a patient to have fever, splenomegaly, cytopenias in at least two cell lines, hypertriglyceridemia and/or hypofibrinogenemia, and histopathologic confirmation of hemophagocytosis in bone marrow or spleen biopsy [19]. Since many patients present with some of these five criteria and develop the rest during the course of their illness, and because early diagnosis and treatment are essential for survival, the HLH-2004 criteria included the same five criteria and added three more: hyperferritinemia, elevated CD25 levels, and reduced NK cell activity, requiring a patient to meet at least five criteria for diagnosis [20].

The formal scoring systems were developed for patients under 18 years of age and for primary HLH. It is possible that the proliferation of predictive models, such as the H-Score and the Optimized HLH Inflammatory Index (OHI), has contributed to higher rates of diagnosis, since these models are widely used in adult populations [20,21]. Both models are based on the HLH-2004 diagnostic criteria and generate a numeric value corresponding to the probability of an underlying HLH diagnosis. The OHI index was developed and validated specifically for patients with underlying hematologic malignancy [22]. Interestingly, recent studies have shown that elevated soluble CD25 and ferritin levels are key markers that can help distinguish hematologic malignancies without HLH from those with HLH [23]. The use of the OHI score has likely contributed to increased recognition in this population, which also carries a poor prognosis compared to other forms of secondary HLH [4]. The overall mortality rate may continue to rise due to higher age at incidence, and similarly, the AAMR may continue to increase with greater awareness and testing, particularly among patients with known hematologic malignancies who have low overall survival.

Strengths and limitations

This study involves an analysis of death records, which rely on accurate diagnostic coding for a condition that, until recent decades, was primarily recognized by specialists. Similar to other studies using CDC WONDER, it benefits from a large sample size and longitudinal trends relevant to the hypothesis being tested. However, without individual-level data, we cannot make definitive claims about the factors driving the increase in D76.1 codes on death records, and our conclusions are therefore inferential, based on population-level data. Although the data exported from CDC WONDER are age-adjusted, they do not automatically account for ethnicity or sex, and underlying mortality trends within each group may mask or exaggerate D76.1-associated mortality trends. We stratify by region, ethnicity, and sex to mitigate this limitation. Finally, although we used the "underlying cause of death" functionality, this depends on clinicians accurately completing death certificates, which we cannot independently verify.

## Conclusions

There has been a significant and consistent increase in AAMR from HLH per 1,000,000 adults in the United States from 2010 to 2023, observed across census regions, ethnicities, and sexes. There has been no corresponding decline in the AAMR of related diseases, suggesting, but not definitively proving, that this trend is independent of changes in coding practices. Instead, it may result from a combination of factors, including improved disease recognition, broader diagnostic criteria, and the increasing age of the general population with concurrent incidence of underlying conditions, such as hematologic malignancies, that can trigger secondary HLH.
